# ATPase Activity and ATP-dependent Conformational Change in the Co-chaperone HSP70/HSP90-organizing Protein (HOP)*

**DOI:** 10.1074/jbc.M114.553255

**Published:** 2014-02-17

**Authors:** Soh Yamamoto, Ganesh Prasad Subedi, Shinya Hanashima, Tadashi Satoh, Michiro Otaka, Hideki Wakui, Ken-ichi Sawada, Shin-ichi Yokota, Yoshiki Yamaguchi, Hiroshi Kubota, Hideaki Itoh

**Affiliations:** From the ‡ Department of Life Science, Faculty and Graduate School of Engineering and Resource Science, Akita University, Akita 010-8502, Japan,; the § Department of Microbiology, Sapporo Medical University School of Medicine, Sapporo 060-8556, Japan,; the ¶ Structural Glycobiology Team, Systems Glycobiology Research Group, RIKEN-Max Planck Joint Research Cluster for Systems Chemical Biology, 2-1 Hirosawa, Wako, Saitama 351-0198, Japan,; the ‖ Department of Gastroenterology, Juntendo University School of Medicine, 2-1-1 Hongo, Bunkyo-Ku, Tokyo, Japan, and; the ** Third Department of Internal Medicine, Akita University School of Medicine, Akita 010-8543, Japan

**Keywords:** Heat Shock Protein, Hsp90, Molecular Chaperone, Protein Complexes, Protein Structure

## Abstract

Co-chaperones help to maintain cellular homeostasis by modulating the activities of molecular chaperones involved in protein quality control. The HSP70/HSP90-organizing protein (HOP) is a co-chaperone that cooperates with HSP70 and HSP90 in catalysis of protein folding and maturation in the cytosol. We show here that HOP has ATP-binding activity comparable to that of HSP70/HSP90, and that HOP slowly hydrolyzes ATP. Analysis of deletion mutants revealed that the ATPase domain of HOP is in the N-terminal TPR1-DP1-TPR2A segment. In addition, HOP changes its conformation in the presence of ATP. These results indicate that HOP is a unique co-chaperone that undergoes an ATP-dependent conformational change.

## Teaser

**Background:** HOP assists protein transfer in the HSP70- and HSP90-dependent protein-folding pathway.

**Results:** HOP hydrolyzed ATP, and the region containing amino acids 1–359 (TPR1-TPR2A) was required for hydrolysis and direct interaction with ATP.

**Conclusion:** HOP has slow ATPase activity and changes its conformation upon ATP hydrolysis.

**Significance:** This is the first demonstration of the ATPase activity of HOP, and may enhance our understanding of the physiological function.

## Introduction

The 70 kDa and 90 kDa heat shock proteins (HSP70 and HSP90)^2^ are major molecular chaperones in the eukaryotic cytosol. These chaperones play essential roles in protein quality control by preventing protein aggregation, catalyzing the folding of newly synthesized proteins, and promoting degradation of denatured proteins (1–3). HSP70 recognizes hydrophobic surfaces of unfolded and partially folded proteins, and it releases these substrate proteins after undergoing conformational changes over the course of its ATPase cycle (4). HSP70 consists of a highly conserved N-terminal nucleotide-binding domain (NBD) and a C-terminal substrate-binding domain (SBD) (5, 6). HSP90 consists of three functional domains, the N-terminal, middle, and C-terminal dimerization domains. The middle domain is involved in ATP hydrolysis, client protein binding, and co-chaperone binding, whereas the C-terminal domain is required for HSP90 dimerization (7, 8). During the ATPase cycle, open and closed structures of HSP90 are produced, and these conformations are important for stabilization of the client proteins (9, 10).

HSP70 and HSP90 cooperate with co-chaperones during the process of protein folding, including maturation of steroid hormone receptors, kinases, and p53 (11, 12). The HSP70/HSP90 organizing protein (HOP, also known as stress-inducible protein 1 or STI1) is a co-chaperone that cooperates with HSP70 and HSP90 in protein folding. HOP acts as a scaffold for HSP70/HSP90 and modulates the functions of these chaperones (12–16). HOP is a monomeric protein (9, 17) composed of three tetratricopeptide repeat domains (TPR1, TPR2A, and TPR2B) and two aspartic acid-proline domains (DP1 and DP2) (16). The TPR domains are protein-protein interaction modules containing helix-turn-helix structures (18). HOP binds HSP70 and HSP90 through the TPR1 domain or the TPR2B domain and TPR2A domain via ionic interactions (19–21). The solution structure of the yeast HOP/STI1 DP domains has been determined by nuclear magnetic resonance (NMR) analysis, which revealed that the DP1 and DP2 domains consist of six and five helices, respectively (21). The DP2 domain provides important support for the chaperone activity of HSP90 (21, 22). However, the detailed mechanisms of HOP-assisted, chaperone-dependent protein folding are largely unknown. Here, we report that HOP has ATPase activity and changes its conformation in the presence of ATP. We discuss the possible roles of ATP-dependent conformational changes in regulating HOP function.

## EXPERIMENTAL PROCEDURES

### 

#### 

##### Expression Vectors and Purification

Expression vector encoding human full-length HOP-(1–543) was constructed as previously described (10). DNAs encoding domain-deletion mutants (containing amino acids 106–543, 352–543, 1–359, 1–224, 106–224, 225–309, and 106–309) were generated by polymerase-chain reaction (PCR) using specific primers, and then subcloned into the pCold I hexahistidine-tagged (His_6_) protein expression vector (Takara, Tokyo, Japan). The resultant vector were transformed into *Escherichia coli* BL21, and the bacteria were grown in L-broth at 37 °C. Expression of His_6_-tagged full-length HOP and its deletion mutants were induced in the presence of 1 mm isopropyl-1-thio-β-d-galactopyranoside for 24 h at 15 °C. Bacteria were harvested, resuspended in 10 mm Tris-HCl buffer, pH 7.4, and lysed by sonication on ice. Supernatant was recovered after centrifugation at 20,000 rpm for 5 min at 4 °C and then applied onto a Ni-NTA column (GE Healthcare, Amersham Biosciences) equilibrated with buffer A (300 mm NaCl, 10 mm Tris-HCl, pH 7.4) supplemented with 20 mm imidazole. After the column was washed with ten column volumes of buffer A containing 50 mm imidazole, proteins were eluted with a linear gradient of 100–500 mm imidazole in buffer A. HOP peak fractions were pooled and concentrated by ultrafiltration. Concentrated HOP solution was loaded onto a Superdex 200 HR column (GE Healthcare) equilibrated with buffer B (5% glycerol, 1 mm DTT, 150 mm NaCl in 25 mm HEPES-KOH, pH 7.4). HOP peak fractions were pooled and dialyzed against buffer C (5% glycerol, 1 mm DTT in 25 mm HEPES-KOH, pH 7.4). Purified HOP was frozen using liquid nitrogen and stored at −80 °C. Purification of HSP70 and HSP90 from porcine brain was performed as described previously (23, 24). Protein concentration was determined by the bicinchoninic acid method using the BCA Protein Assay kit (Pierce) and bovine serum albumin as the standard.

##### Measurement of ATPase Activity and Kinetic Analysis

HOP (20 μm), porcine HSP70 (2 μm), and porcine HSP90 (4 μm) were incubated with ATP in buffer D (1 mm DTT, 5 mm MgCl_2_, 50 mm KCl in 25 mm HEPES-KOH, pH 7.4) at 37 °C for 90 min. All proteins were treated as monomers for purposes of calculating concentration. The amount of hydrolyzed ATP was determined as previously described (24). Kinetic parameters were calculated by non-linear regression using the Michaelis-Menten equation and the Origin 6.1 software (Originlab, Northampton, MA). To determine ATPase activity, domain-deletion mutants (20 μm) were incubated with ATP in buffer D at 37 °C for 120 min.

##### Protease Sensitivity Test

HOP (4 μm) was suspended in buffer E (5 mm MgCl_2_, 100 mm KCl in 25 mm HEPES-KOH, pH 7.4) and digested with 10 nm TPCK-trypsin and 30 nm proteinase K for 30 min at 37 °C in the presence of 5 mm nucleotides. After incubation, partially digested HOP fragments were separated by 10% sodium dodecyl sulfate-polyacrylamide gel electrophoresis (SDS-PAGE) followed by Coomassie Brilliant Blue staining. Band intensities were analyzed by the public-domain ImageJ software (US Natl. Inst. Health).

##### Determination of the N-terminal Amino Acid Sequence

The protease-treated samples were separated by SDS-PAGE on 11% gels and blotted onto polyvinylidene difluoride filters. After blotting, the filter was stained with 0.025% Coomassie Brilliant Blue R250 in 40% ethanol for 30 min at room temperature, de-stained by washing in 50% ethanol, and washed in 100% ethanol. Protein bands excised from filters were N-terminally sequenced using a LC 491 protein sequencer (Applied Biosystems).

##### NMR Spectrometry

NMR experiments were recorded using a 600 MHz spectrometer (DRX-600, Bruker Biospin) equipped with a 5 mm triple-resonance inverse (TXI) probe or with a 5 mm BBO (Broadband Observe) probe. For monitoring of protein signals, proteins (0.3 mm) were dissolved in 20 mm potassium phosphate buffer (pH 6.5), 50 mm KCl, and 5 mm MgCl_2_ containing 10% (*v*/*v*) D_2_O, and then titrated ATP at a 10-fold molar excess. Probe temperature was set at 25 or 5 °C, and the water signal was suppressed using the WATERGATE pulse sequence.

For determination of dissociation constants, all the samples were prepared in 25 mm MES and 5 mm MgCl_2_, pH 6.5. For the titration experiment, HOP was used at a concentration 135 μm. Various amounts of ATP stock solutions were added to HOP solutions, and ^1^H and ^31^P NMR spectra of ATP were recorded after each titration. To determine the dissociation constant (*K_d_*) of HOP with ATP, the chemical shift changes of H2 (8.25 ppm) and H8 (7.88 ppm) protons of ATP were taken, because these proton signals exhibited chemical shift changes as the ATP concentration was gradually increased. NMR data were processed using XWIN-NMR ver. 3.5 (Bruker Biospin).

*K_d_* values were determined by a non-linear fitting method using Equation 1.

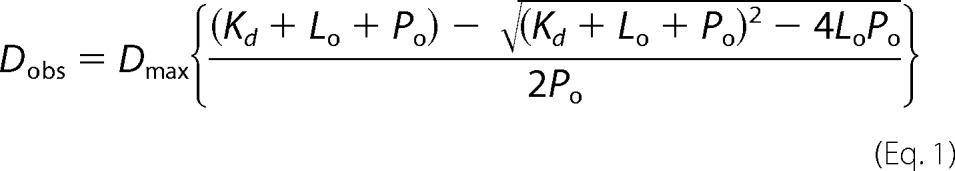
 Here, *L*_o_ and *P*_o_ indicate the molar concentrations of HOP and ATP, respectively; *D_obs_* is the observed chemical shift change; and *D_max_* is the change in chemical shift at saturation.

## RESULTS AND DISCUSSION

### 

#### 

##### HOP Binds ATP with an Affinity Comparable to That of HSP70 or HSP90 and Slowly Hydrolyzes ATP

**FIGURE 1.**
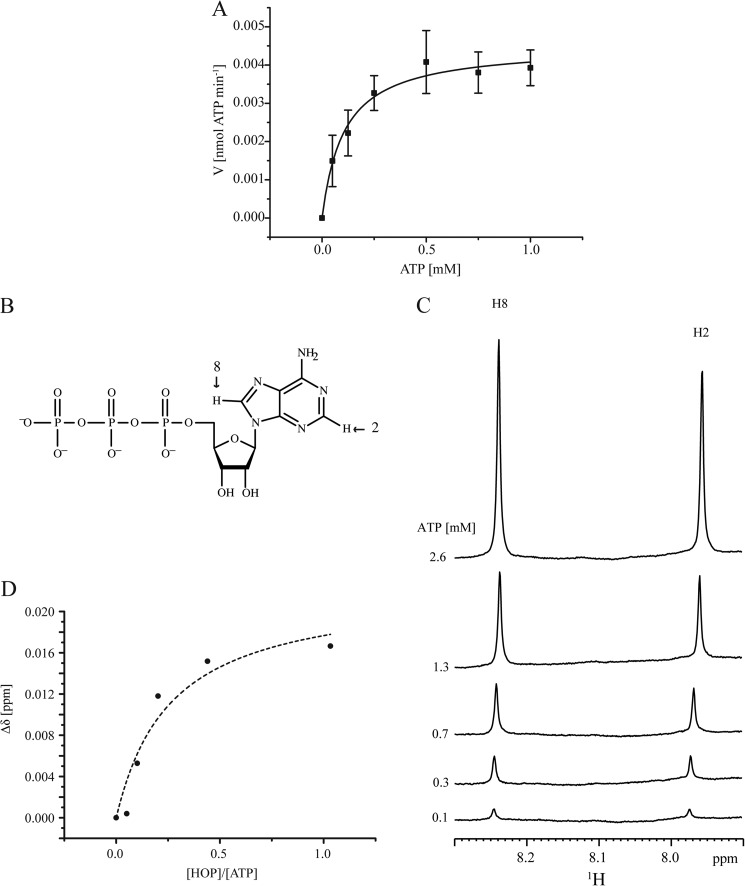
**HOP has ATPase activity.** *A*, plot of the HOP ATP hydrolysis rate *versus* ATP concentration. The kinetic parameter was calculated by non-linear regression using the Michaelis-Menten equation. Means and standard deviations (S.D.) from at least three independent measurements are shown. *B*, structure of ATP. *Arrows* indicate the determined positions of the H2 and H8 protons of ATP. *C*, ^1^H-NMR spectra of ATP titration with HOP at 5 °C. *D*, plot of the change in chemical signal of protons (in ppm) *versus* molar ratio of ATP and HOP. Average of the H2 and H8 proton chemical shifts is shown. The *broken line* indicates theoretical curves obtained using the equation described under “Experimental Procedures.”

Using purified HOP expressed in *Escherichia coli*, we analyzed the ATPase activity of the purified HOP and determined the kinetic parameters using the Michaelis-Menten equation (Fig. 1*A*). The catalytic constant (*k_cat_*), which represents the number of ATP molecules hydrolyzed by HOP, was estimated to be 3.8 ± 0.3 × 10^−3^ mol ATP/mol HOP/min. The ATP hydrolysis rate of HOP was lower than those of porcine brain HSP70 (*k_cat_* = 85.3 ± 15.0 × 10^−3^) and HSP90 (*k_cat_* = 27.9 ± 3.4 × 10^−3^). To determine the dissociation constant (*K_d_*) of HOP for ATP, we performed NMR titration experiments. We first examined the ^31^P chemical shift changes of the ATP phosphate group; no significant changes were observed (data not shown). Therefore, we analyzed the chemical shift changes on adenine H2 and H8 protons of ATP. We observed chemical shift changes in both the H2 and H8 proton signals at each titration point (Fig. 1*C*). These chemical shift changes were used for the determination of *K_d_* values using non-linear fitting methods, and *K_d_* was estimated to be 350 μm (Fig. 1*D*). These results indicated that HOP binds to ATP with considerable affinity and then slowly hydrolyzes ATP.

**FIGURE 2.**
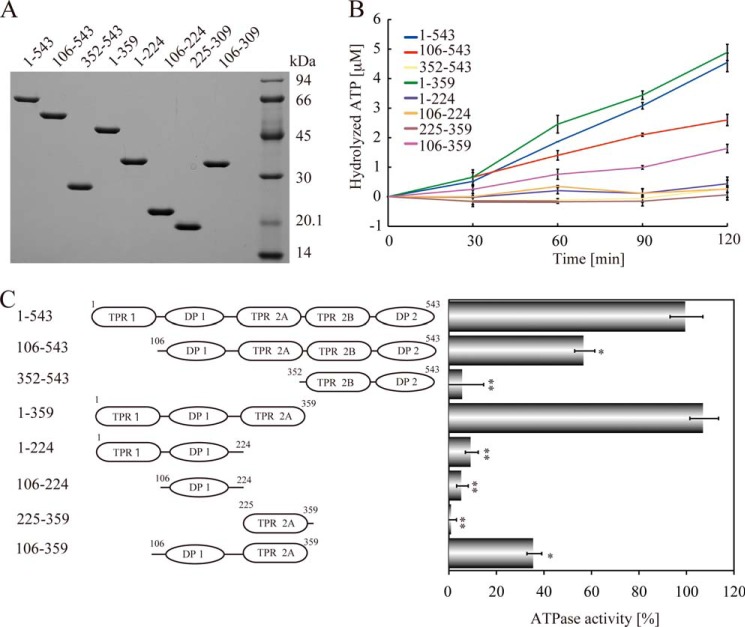
**The 1–359 region of HOP is the central domain responsible for ATPase activity.** *A*, purified deletion mutants of human HOP (1.5 μg/lane) were separated by SDS-PAGE. *B*, HOP mutants (20 μm each) were incubated with 0.5 mm ATP for 120 min, and hydrolyzed ATP (μm) was determined. *C*, relative ATPase activities of HOP deletion mutants. HOP deletion mutants were incubated with 0.5 mm ATP. The ATPase activity of HOP-(1–543) (full-length) is defined as 100%. Means and S.D. from at least three independent experiments are shown. *, *p* < 0.01; **, *p* < 0.001.

To determine the domains responsible for the ATPase activity, we constructed a series of deletion mutants and purified the protein segments from bacteria (Fig. 2*A*). These segments were incubated with ATP at 37 °C for up to 120 min, and then ATP hydrolysis activities were determined (Fig. 2, *B* and *C*). The ATPase activity of the HOP-(1–359) fragment (TPR1-TPR2A) was very similar to that of full-length HOP-(1–543), defined here as 100%. By contrast, the 106–543 (ΔTPR1) and 352–543 (TPR2B-DP2) fragments exhibited significantly reduced ATPase activities (57.2 ± 4.3% and 6.1 ± 8.5%, respectively). These results indicated that the 1–359 region plays an important role in ATPase activity. We further analyzed the 1–359 region by analyzing smaller sub-fragments. The ATPase activity of the 106–359 fragment (DP1-TPR2A) was 36.0 ± 3.1%, and the ATPase activities of the 1–224 (TPR1-DP1), 106–224 (DP1 domain), and 225–359 (TPR2A domain) fragments were almost negligible (9.6 ± 2.7%, 5.7 ± 2.5%, and 1.4 ± 1.8%, respectively). These results indicated that the 106–359 region is essential for the ATPase activity of HOP, where the 1–105 region makes a significant, but smaller contribution to ATPase activity.

**FIGURE 3.**
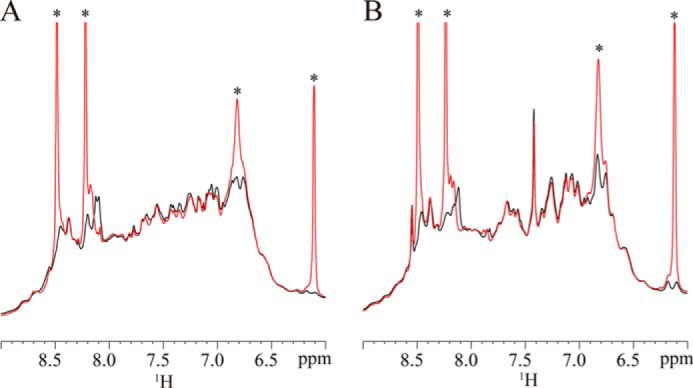
**^1^H-NMR spectra (amide region) of HOP-(1–543) and HOP-(1–359) titrated with ATP.** ^1^H-NMR spectra of 0.3 mm full-length HOP-(1–543) (*A*) and 0.3 mm HOP-(1–359) (*B*) measured at 25 °C in the absence (*black*) or presence of 10-fold molar excess of ATP (*red*). Signals marked with *asterisk* are derived from ATP.

We next used NMR spectroscopy to determine whether ATP directly interacts with the ATPase domain of HOP (Fig. 3). Specifically, we obtained the ^1^H NMR spectra of full-length HOP and the 1–359 region containing the ATPase domain (TPR1-TPR2A). ^1^H chemical shift changes were observed for full-length HOP and the ATPase domain in the presence of ATP. These data indicated that ATP directly binds the ATPase domain of HOP.

**FIGURE 4.**
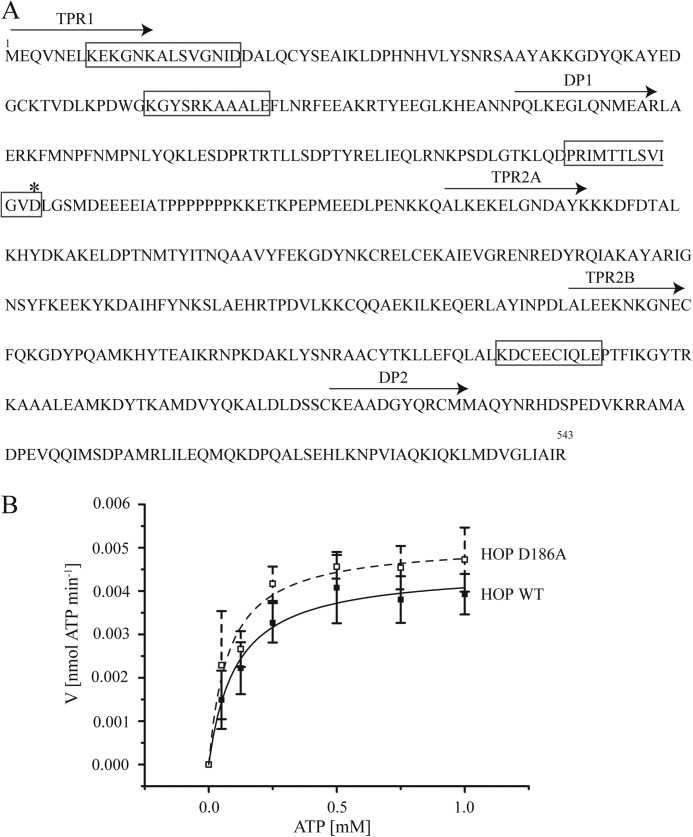
**ATPase activity of HOP is unaffected by the D186A mutation.** *A*, *arrows* indicate the starting points of domains, and *gray boxes* represents Walker B motif. *Asterisk* shows the mutated aspartic acid residue at position 186. *B*, plot of ATP hydrolysis rate *versus* ATP concentration. Activities of wild-type HOP and the D186A mutant are indicated by *black* and *broken lines*, respectively. Means and S.D. from at least three independent measurements are shown.

To identify the ATP-binding site of human HOP, we searched the amino acid sequence of HOP for known ATP-binding motifs, *i.e.* the Walker A motif (G/A*XXXX*GK(*X*)S/T, where *X* indicates any amino acid), also know as the P-loop and phosphate-binding motif (25) and the Walker B motif (R/K-*X*_2–10_-O-*X*-O-D/E, where O indicates a hydrophobic amino acid) (26). The Walker B motif binds a divalent ion and the adenosine residue of ATP, and this motif is present in HSP70, HSP90, and other chaperones (27). HOP contains a Walker B motif in the TPR1, DP1, and TPR2B domains (Fig. 4*A*), but no Walker A motif. We focused on the Walker B motif of the DP1 domain because this region was essential for ATP hydrolysis (Fig. 3, *B* and *C*). To disrupt the Walker B motif, we constructed the D186A mutant in DP1. However, this mutation caused no significant change in ATPase activity (Fig. 4*B*). These results indicate that the Asp-186 residue is not essential for HOP ATPase activity, and suggest that HOP binds ATP using motifs other than Walker A and B. The NBD of the HSP70 and HSP90 structures are similar to those of actin and DNA-gyrase B, respectively (5, 28), and the ATP hydrolysis mechanisms of these non-Walker ATPases were recently elucidated (4, 8, 12, 21). Another non-Walker ATPase is the chaperonin GroEL; this chaperone has a unique ATP-binding motif (DGTTT) and phosphate-recognition region, *i.e.* a P-loop arrangement located between two α-helices (25, 29). Thus, HOP may contain non-Walker ATP-binding motifs, although these motifs remain to be determined.

##### HOP Changes Its Conformation in the Presence of ATP

**FIGURE 5.**
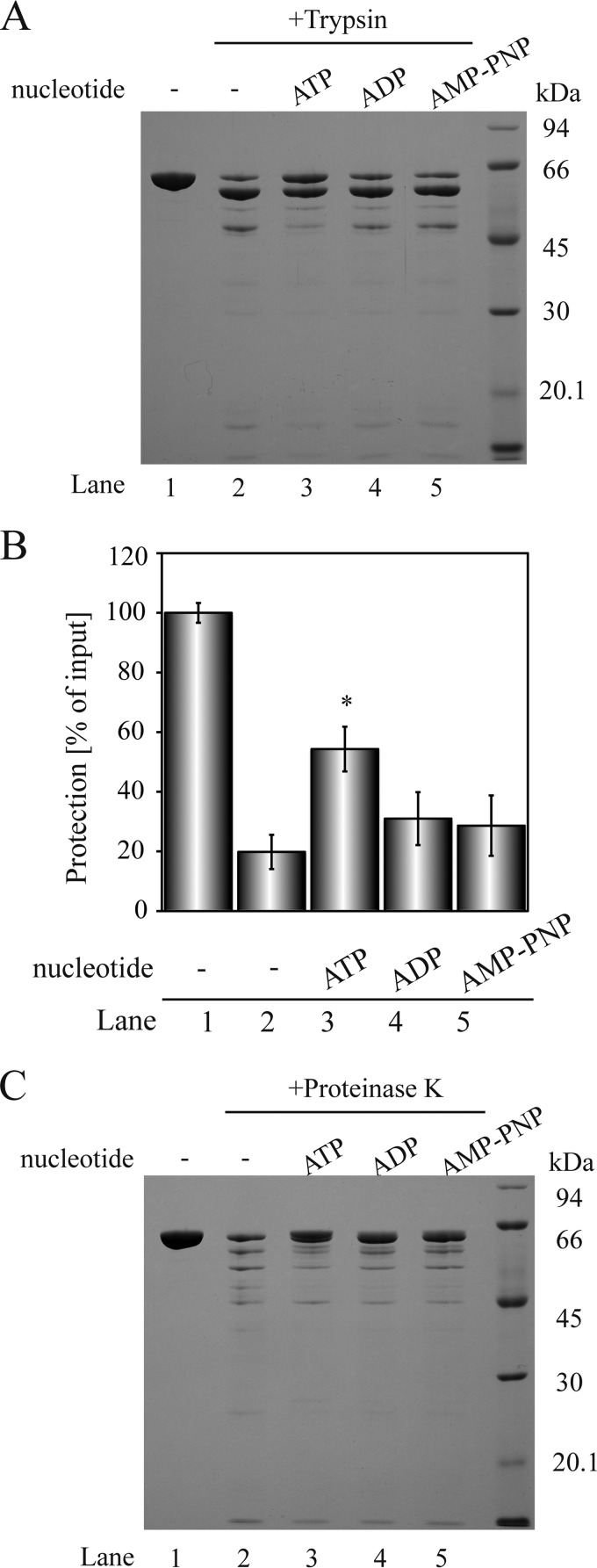
**HOP changes its conformation in the presence of nucleotides.** *A*, HOP (3 μm) was incubated for 30 min at 37 °C with or without TPCK-trypsin (10 nm) in the presence of nucleotides (5 mm), and then separated by SDS-PAGE. *B*, band intensity of undigested HOP was quantified from three independent measurements. *, *p* < 0.05. *C*, HOP (3 μm) was incubated for 30 min at 37 °C with or without proteinase K (30 nm) in the presence of nucleotides (5 mm), and then separated by SDS-PAGE.

The ^1^H chemical shift change of full-length HOP and the 1–359 region containing the ATPase domain suggested that HOP might undergo conformational changes upon ATP binding (Fig. 3). In addition, most molecular chaperones change their conformations during ATP binding and hydrolysis (8). To determine whether HOP exhibits ATP-dependent conformational changes, we first performed protease digestion assays (30). HOP was incubated with trypsin in the presence and absence of various nucleotides (Fig. 5). HOP was more effectively protected from trypsin digestion in the presence of ATP (Fig. 5*A*, *lane 3*) than in the presence of ADP and AMP-PNP, a non-hydrolyzable analog of ATP; the protection efficiency was 54.3 ± 7.5% for ATP, 31.0 ± 8.9% for ADP, and 28.6 ± 10.1% for AMP-PNP, respectively (Fig. 5*B*). Furthermore, the proteinase K digestion experiment suggests that the conformation of HOP in the presence of ATP differs from that in the presence of ADP or AMP-PNP, because the proteinase K-digested HOP fragments produced in the presence of ATP were partly different from those produced in the presence of ADP or AMP-PNP (Fig. 5*C*). Although HSP70 and HSP90 change their conformations upon nucleotide binding and hydrolysis (8, 31), the chaperonin CCT/TRiC changes its conformation only during ATP hydrolysis but not upon nucleotide binding (30). These observations suggest that the role of HOP in the ATP-dependent conformational changes may be similar to that of CCT/TRiC, but not to those of HSP70 and HSP90.

We next investigated the effect of ATP on the secondary structures of HOP by analysis of circular dichroism (CD) spectra. However, no significant change was detected (data not shown). Taken together with the results of the NMR and protease digestion experiments (Figs. 3*A* and 5), these results suggest that in the presence of ATP, HOP changes the relative orientations, but not the secondary structures, of its domain.

**FIGURE 6.**
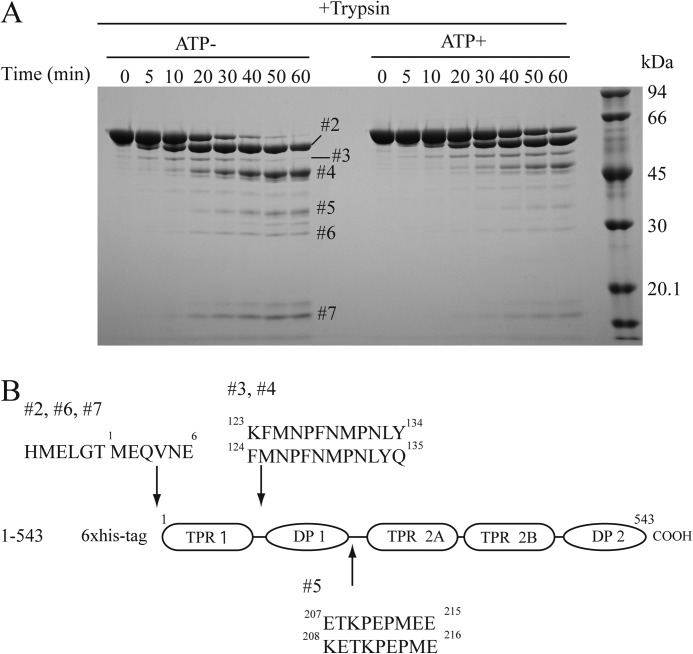
**The ATP-dependent conformational change of HOP occurs throughout the entire HOP molecule.** *A*, time course of TPCK-trypsin digestion of HOP. After incubation with or without ATP, samples were analyzed by SDS-PAGE. *B*, N-terminal amino acid sequences obtained using a protein sequencer are shown; *numbers* indicate the corresponding bands (see *panel A*).

To further analyze the regions affected by the ATP-dependent conformational changes, we determined the amino acid sequences of trypsin-digested HOP fragments (Fig. 6, *A* and *B*). This sequencing analysis revealed that the region between TPR1 and DP1 was sensitive to protease digestion. Similarly, the hinge region between DP1 and TPR2A was susceptible to enzymatic cleavage. Because, the relative proportions of the fragments produced by digestion at these cleavage sites (#3, #4, and #5) changed in the presence of ATP, these finding indicated that the structure of HOP is altered, at least somewhat, at the N-terminal region near the DP1 domain. However, the level of the large #2 fragment rose in the presence of ATP, suggesting that the conformation of the C-terminal region is also affected by ATP. Thus, the conformational change at the N-terminal ATPase domain may trigger structural alteration of the C-terminal domain, although this possibility remains to be conclusively demonstrated. Nevertheless, these observations support the notion that the ATP-dependent conformational change of HOP occurs throughout the entire HOP molecule, rather than in a small restricted region.

HOP is a multi-domain protein containing three TPR and two DP domains. The TPR domains are protein-interaction modules that act as a scaffold for the HSP complex. TPR1 and TPR2B bind to HSP70, whereas TPR2A binds to HSP90 (19, 21). Recently, the TPR1-DP1 segment was suggested to serve as an HSP70-client delivery system for the TPR2A-TPR2B-DP2 segment, because HSP70 changes the HOP binding domain from TPR1 to TPR2B during the maturation of substrate proteins (21). The role of the DP1 domain is not well understood (32, 33). By contrast, the DP2 domain is known to be essential for HSP90-assisted protein maturation, because mutants lacking the DP2 domain are unable to assist in glucocorticoid receptor activation (21, 22, 34). In this study, we demonstrated that HOP changes its conformation in the presence of ATP. We also revealed that the central ATPase domain is localized at the DP1-TPR2A segment, and that the TPR1 domain plays an additional role in the ATPase activity. Taken together with the fact that HOP changes its conformation upon HSP90 binding, these observations suggest that the ATP-dependent conformational change of HOP may contribute to HSP70/HSP90-assisted protein folding and maturation by rearranging the orientations of the five domains of HOP (35).

HOP, which is ubiquitously expressed in all cell type, and localizes in the cytoplasm, nucleus, and endoplasmic reticulum, as well as on the cell surface (33, 36–41). Transportation of HOP from the cytoplasm to the nucleus is regulated by phosphorylation (41). HOP affects maturation of the cystic fibrosis transmembrane conductance regulator protein through *S*-nitrosylation (37). In addition, HOP functions in neuroprotection and neuritogenesis by interacting with the prion protein on the cell surface (40, 42). These studies suggest that HOP has a variety of functions *in vivo*, in addition to its co-chaperone function for HSP70 and HSP90.

Based on our finding, we conclude that, cooperation between the N-terminal ATPase domain and the C-terminal chaperone interacting domain may be required for co-chaperone activity of HOP or other HOP-dependent cellular functions.

## Footnotes

2 The abbreviations used are:

HSP: heat shock protein
HOP: HSP70/HSP90-organizing protein
NBD: nucleotide-binding domain
SBD: substrate-binding domain
AMP-PNP: adenosine 5′-(β,γ-imino)triphosphate.
